# The use of apatinib in treating primary pleural synovial sarcoma: A case report

**DOI:** 10.1097/MD.0000000000018382

**Published:** 2019-12-20

**Authors:** Sumei Chen, Ke Zhang, Xianqin Wan, Yuanyuan Chen, Shenglin Ma, Qinghua Deng

**Affiliations:** a Department of Radiation Oncology, Hangzhou Cancer Hospital; b Hangzhou First People's Hospital, Affiliated to Zhejiang University, Hangzhou, Zhejiang, China.

**Keywords:** apatinib, pleural synovial sarcoma, soft tissue sarcomas, VEGFR-2

## Abstract

**Rationale:**

Apatinib is an oral tyrosine kinase inhibitor targeting vascular endothelial growth factor receptor-2. It has been shown that apatinib is effective and safe for treatment of multiple solid tumors, including gastric cancer, liver cancer, non-small-cell lung cancer, and breast cancer. However, there is currently no consensus as to using Apatinib for the treatment of pleural synovial sarcoma, due to the rarity of primary pleural synovial sarcoma and lack of clinical studies as a consequence.

**Patient concerns and diagnoses:**

We reported here in the case of a 26-year-old Chinese woman diagnosed with pleural synovial sarcoma. She has undergone 2 surgeries, multiple regimens of chemotherapy and traditional Chinese medicine in other hospitals. Then the patient was admitted to our hospital with the compliant of chest pain and dyspnea. The medical history and available data supported the diagnosis of recurrence of pleural synovial sarcoma.

**Interventions and outcomes:**

Due to the lack of efficacy of previous standard treatment, the patient was given apatinib and radiotherapy to relieve the symptoms. This patient achieved stable disease with apatinib at a dose of 500 mg/day. Her progression-free survival time was more than 7 months, and her overall survival was 8.5 months. Except for hand-foot syndrome, no grade 3 or 4 side effects were observed.

**Conclusions:**

Apatinib may thus be an option for treatment of advanced synovial sarcoma after failure of other treatments. However, further study is needed to determine the efficacy of apatinib in pleural synovial sarcoma.

## 1 Introduction

Synovial sarcomas are one subtype of soft tissue sarcomas accounting for approximately 1% of all adult malignancies.^[1]^ Synovial sarcomas are rare and aggressive, and most commonly affect extremities near large joints in young adults. They also occur in other parts of the body, including head and neck, lung, pleura, mediastinum, mesentery, and intra-abdominal, and pelvic spaces. Primary pleural synovial sarcoma is extremely rare, with only about 40 cases reported so far since the disease was first documented by Gaertner in 1996.^[2]^

The 5-year survival rate for synovial sarcoma ranges from 0% to 88%.^[3]^ Histologic subtype and grade, patient age, tumor size, location, clinical stage, vascular invasion, and pathologic resection margins are all significant prognosis predictors.^[4,5]^ Because of its low incidence and the lack of clinical studies, there is currently no consensus as to the optimal therapy for pleural synovial sarcoma. Surgical resection is the only potentially curative treatment for localized pleural synovial sarcoma.^[6]^ Unfortunately, by the time most patients are diagnosed, the disease is too advanced and opportunity for surgery is lost.^[6]^ Moreover, even localized pleural synovial sarcomas have a high recurrence rate and usually become refractory to treatment.^[7]^ The disease-free interval for pleural synovial sarcoma is 2 to 14 months after surgical resection. The benefits of adjuvant radiochemotherapy for primary synovial sarcoma of the pleura is unclear, since there are no randomized controlled trials.^[8]^ A 5-year disease-free period after radical multidisciplinary therapy has been reported in 20.9% of cases.^[9]^

Angiogenesis is a hallmark of solid tumors. Since Folkman put forward the idea of anti-tumor angiogenesis therapy, numerous drugs targeting vascular endothelial growth factors (VEGFs) or VEGF receptors (VEGFRs) have been explored.^[10]^ For example, the VEGF-A antagonist bevacizumab has been successfully used for anti-tumor angiogenesis therapy in various types of cancers.^[11–13]^ Apatinib is a novel, orally bioavailable small-molecule tyrosine kinase inhibitor selective for VEGFR-2, which has shown significant beneficial effects in the treatment of a variety of solid tumors including soft tissue sarcomas.^[14–19]^ However, there is not any report of using apatibnib for the treatment of primary pleural synovial sarcoma. We herein report a case of primary pleural synovial sarcoma treated with apatinib after the failure of multiple therapies.

## 2 Ethical statement and consent

Informed written consent was obtained from the patient's elder sister for publication of this case report and accompanying images. Ethical committee approval was waived because of unnecessity. Since the information of this case was collected retrospectively after patient's decease, there was no adverse effect on the patient caused by this study.

## 3 Case presentation

**Figure 1:**
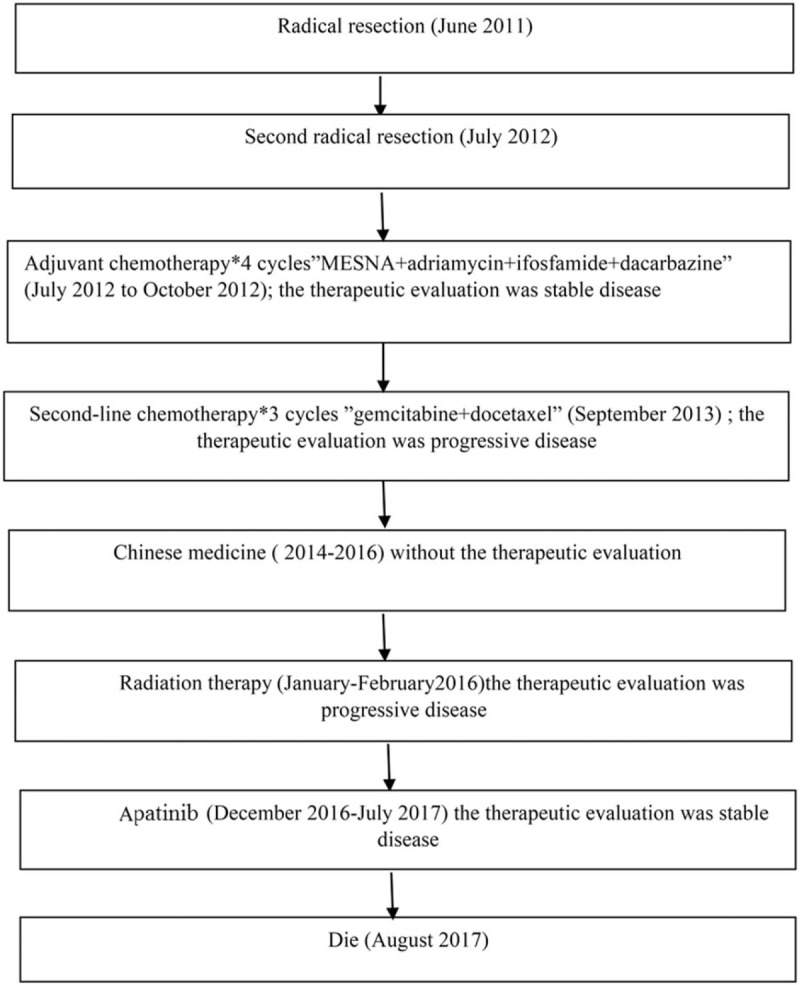
The treatment process for this patient.

A 26-year-old Chinese woman was admitted to the hospital in June 2011 because of repeated pneumothorax. Chest x-rays showed a right sided hydropneumothorax and an area of ill-defined soft tissue density in the lower half of the right lung. Computed tomography (CT) showed a nodule with the size of 4 × 4 centimeter (cm)^2^ in the right lower mediastinum. Exploratory thoracotomy was performed. The treatment process for this patient was shown in Figure 1.

The first radical resection pathological margin was negative, and the minimal resection margin was 5 cm. The postoperative pathological diagnosis was spindle cell synovial sarcoma with the following immunohistochemical features: CD99(+), vimentin (+), Bcl-2 (+), HBME1(−),WT-1 (+), TTF1(−), Napsin A (−), S-100 (−), CK (−), p16 (−), EMA (partly +), NSE (−), Chromogranin A (−). The patient failed to provide immunohistochemistry pictures. The patient received no postoperative chemotherapy or radiotherapy.

**Figure 2:**
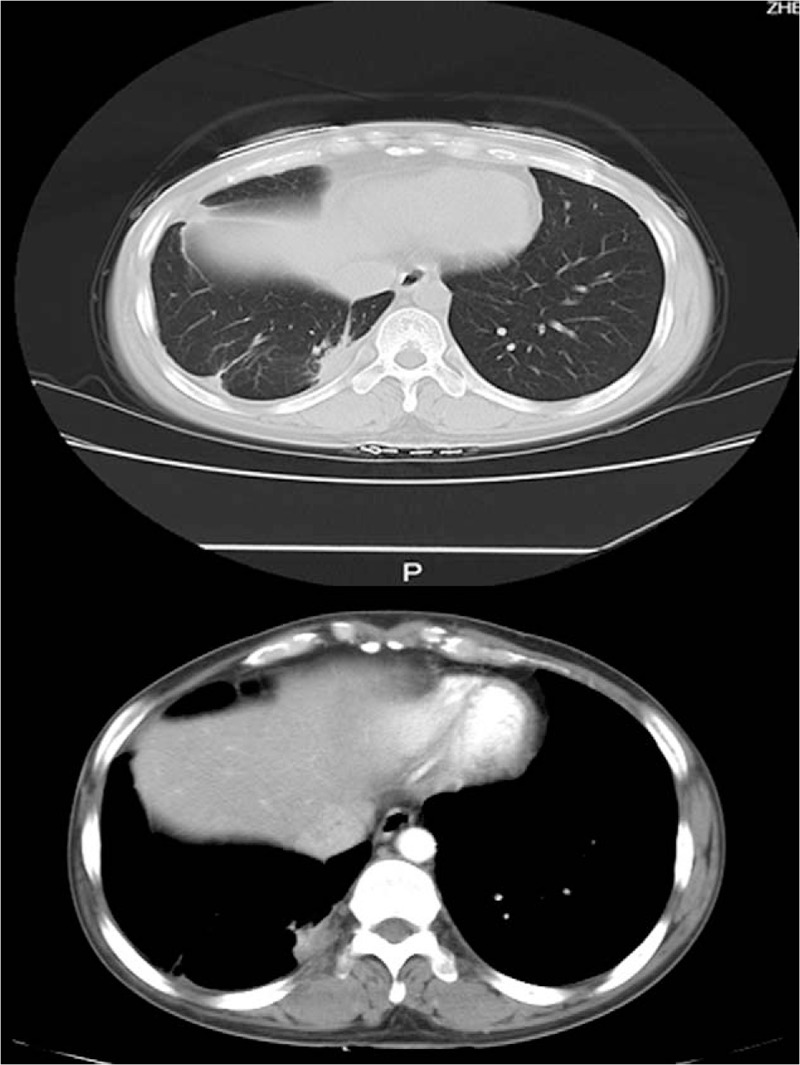
Computed tomography showed that mass in the right pleura.

**Figure 3:**
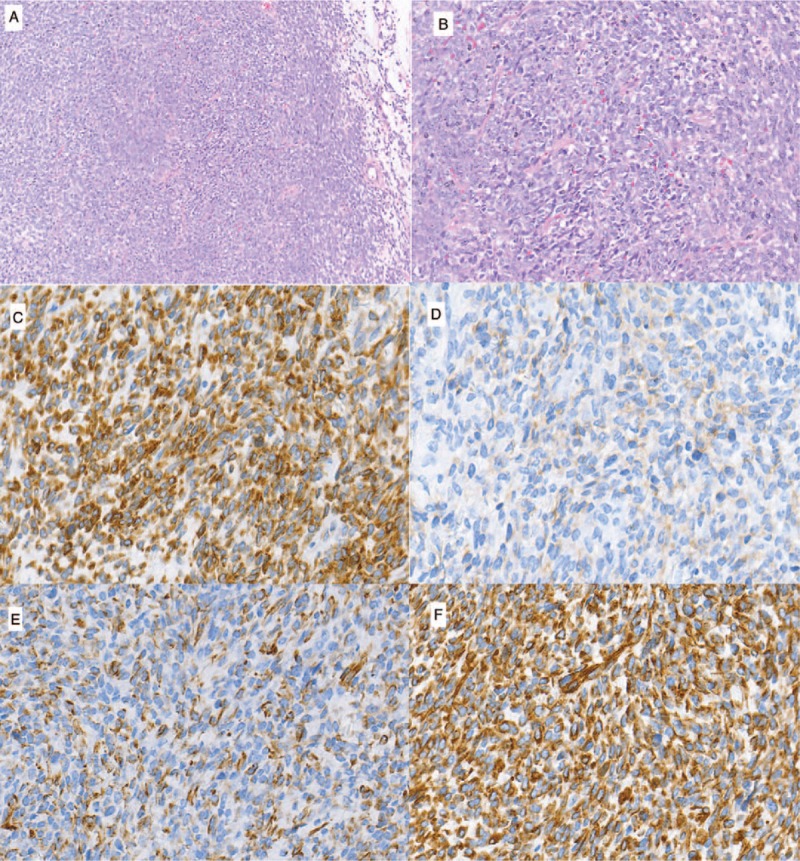
An analysis of the surgical specimen revealed a spindle cell tumor (A and B). A: Hematoxylin and Eosin (H&E) staining (100 × ), A2: H&E staining (200 × ). The immunohistochemical staining was positive for Bcl-2 (C) and CD99 (D) and WT-1(E) and vimentin(F) (400X).

One year later, chest CT and Positron emission tomography/computed tomography (PET/CT) revealed recurrent tumors within the right chest. The chest CT was showed in Figure 2. She was again treated surgically in July 2012, and the pathological margin was negative, but the minimal resection margin was less than 5 cm. The postoperative pathological diagnosis was reoccurrence of synovial sarcoma with the following immunohistochemical features: CD99 (+), vimentin (+), WT-1 (+), CK (−), HBME1 (−), TTF1 (−), Napsin A (−), p16 (−), NSE (−), Chromogranin A (−), Bcl-2(+) (Fig. 3).

**Figure 4:**
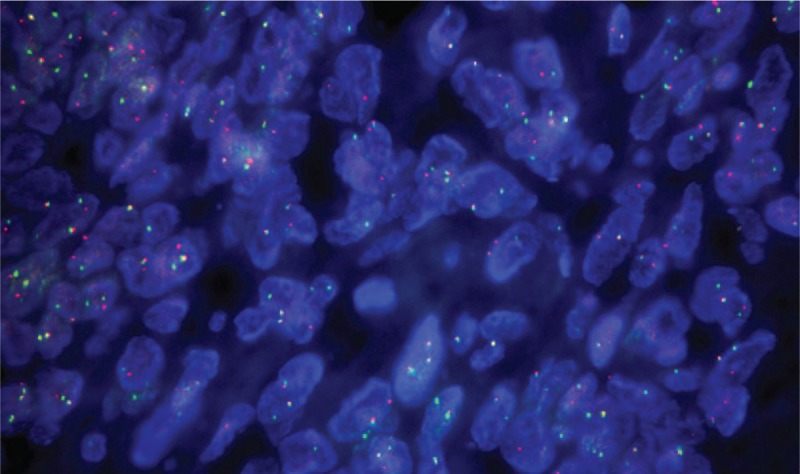
Cytogenetic analysis and FISH resulted from the chromosomal translocation t(X;18)(p11.2;q11.2).

The SYT-SSX fusion gene is the hallmark of synovial sarcoma. Cytogenetic analysis and fluorescent in situ hybridization revealed chromosomal translocation at t(X;18)(p11.2;q11.2) (Fig. 4). During the following 4 years (2012–2016), the patient was administrated with multiple-line therapies, including chemotherapy, radiotherapy, Chinese medicine, and target therapy.

**Figure 5:**
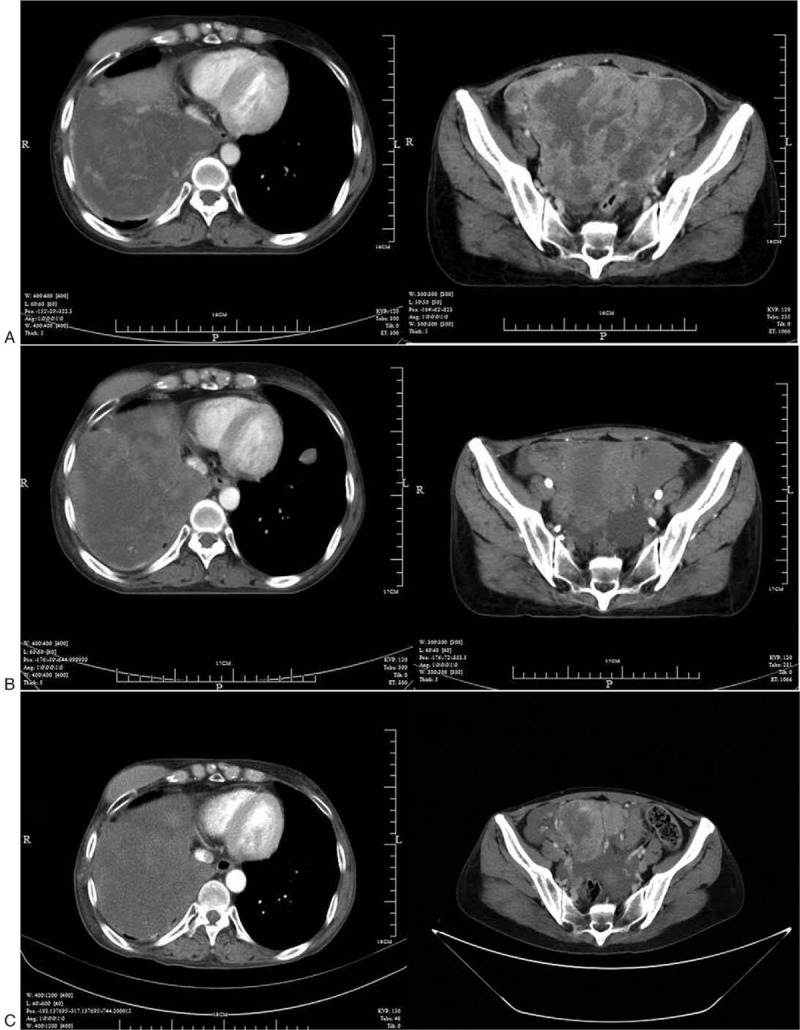
Computed tomography showed huge masses in the right lower chest and pelvis before taking apatinib (A); Computed tomography showed the size of mass following treatment with apatinib of 500 mg daily for 3 months (B); Computed tomography showed the size of mass after taking apatinib for 6 months (C).

**Figure 6:**
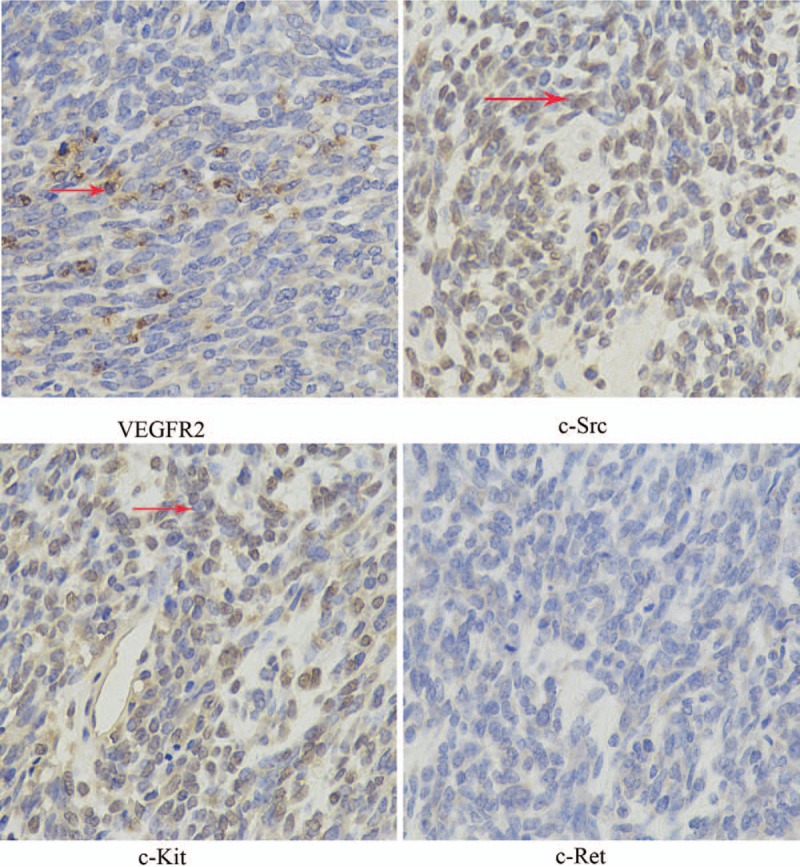
Immunohistochmeistry showing expressions of VEGFR-2, c-Ret, c-Src, and c-Kit proteins (400X), the red arrows point at the positive protein.

Suffering with chest pain and dyspnea, the patient was admitted to our hospital in March 2016. Chest and abdominal CT revealed large masses in the lower right chest and extending to the liver, raising the possibility of metastasis there. We administered a total of 45Gy of intensity modulated radiation therapy, which made the symptoms disappear. But the patient refused systematic treatment and was discharged after completion of radiation therapy. The patient visited our hospital again with serious abdominal pain (NRS 7) and constipation fatigue in December 2016. Figure 5A shows the extensive soft tissue masses with heterogeneous density within the chest, abdominal, and pelvic regions. Tumor progressed after multiline treatment, there is no further standard treatment at present. After the patient provided written, informed consent, oral apatinib (500 mg/d) was administered on December 6, 2016. We also assessed expression of VEGFR-2, c-Ret, c-Src and c-Kit. Of those, VEGFR-2, c-Src and c-Kit were all overexpressed, while c-Ret was negative (Fig. 6). After 3 months of treatment (March 26, 2017), the abdominal and pelvic masses was slightly smaller, and the therapeutic evaluation was stable disease (SD) (Fig. 5B). After 6 months (July 10, 2017), the size of some of the masses had decreased further, and some of metastatic lesions were stable (Fig. 5C). Tumor density gradually decreased, and necrosis increased. The patient developed an outbreak of the tumor more than 7 months after taking apatinib. Since the patient's general condition after tumor progress was very poor, we did not escalate the dosage of apatinib. At last the patient gave up anticancer treatments. A progression-free survival time of more than 7 months was achieved, and overall survival was 8.5 months.

Treatment-related side effects were monitored every 2 weeks during the apatinib treatment. After 2 months, the patient experienced mainly non-hematological toxicities, including skin rash (grade 3), elevated transaminase (grade 1), and proteinuria (grade 1). Thrombocytopenia (grade 2) was detected after 3 months of apatinib treatment. These side effects were controlled with supportive treatment.

## 4 Discussion

As far as we know, this is the first report of using apatinib to treat a patient with reoccurrence of pleural synovial sarcoma. Apatinib is a potent and selective multi-targeted receptor tyrosine-kinase inhibitor. Ji et al^[20]^ reported a case of advanced malignant fibrous histiocytoma in which the patient treated with apatinib exhibited a partial response and progression-free survival for >6 months. They also reported a case of angiosarcoma in which apatinib provided 12 months of progression-free survival.^[21]^ In our case, the patient achieved more than 7 months, progression-free survival time and 8.5 months overall survival. We initially increased the dose of apatinib from 250 mg/d to 850 mg/d gradually and stayed at the dose of 500 mg/d for XXX, which was well tolerated with the minor adverse effect of skin rash (grade 3), elevated transaminase (grade 1), proteinuria (grade 1) and thrombocytopenia (grade 2). These results indicate that apatinib may be a treatment option for patients with advanced synovial sarcoma.

Apatinib may thus be a useful new choice for patients with primary pleural synovial sarcomas. Apatinib (YN968D1, N-[4-(1-cyano-cyclopentyl) phenyl]-2-(4-pyridylmethyl) amino-3-pyridine carboxamide mesylate) is one of the latest orally antiangiogenic agents with attractive pre-clinical and clinical data on the treatment of various solid tumors. However, the exact molecular mechanism of the anticancer effects of apatinib for sarcomas is not well defined. As a small-molecule tyrosine kinase inhibitor, apatinib selectively binds to and strongly inhibits VEGFR-2. Angiogenesis is essential for the growth and progression of solid tumors, and inhibition of VEGF signaling, which plays a key role in angiogenesis, has been a promising anticancer approach. Moreover, VEGF signaling is regulated by a variety of activators and inhibitors,^[22–24]^ which also represent potential targets for anti-angiogenesis therapy. VEGF promotes angiogenesis by stimulating vascular endothelial cells to migrate towards and into hypoxic zones within tumors while protecting them from apoptotic death during the migration.^[24–26]^ VEGFRs (VEGFR-1, -2 and -3) are tyrosine kinases that function as key regulators of this process. VEGFR-2 promotes endothelial proliferation by activating signaling in the mitogen-activated protein kinase pathway during angiogenesis.^[27]^ Overexpression of VEGF/VEGFRs increases the tumor cell proliferation and migration and metastasis. It is thought that by inhibiting VEGF binding to VEGFR-2 and the receptor's subsequent autophosphorylation, apatinib suppresses endothelial cell proliferation, and angiogenesis.^[28]^ It may also inhibit VEGFR-2-mediated down stream phosphorylation of extracellular signal-regulated kinase (ERK), resulting in antiangiogenic and anticancer effects.^[28]^ VEGFR-2 level in the tumor tissue of our patient was overexpressed, which might be the main reason for the anticancer effect of apatinib.

In addition, Tian et al^[29]^ observed that apatinib impaired VEGF-stimulated proliferation, migration and tube formation by human umbilical vein endothelial cells, and blocked rat aortic ring budding in vitro, which may be associated with suppression of VEGFR-2-mediated phosphorylation of Ret, c-kit and c-src. We also tested expression of Ret, c-Src and c-Kit and found that c-Src and c-Kit were overexpressed in our patient. These overexpressed proteins may be related to anticancer effect of apatinib. In other words, VEGFR-2, Ret and c-kit may account for the positive result in our patient.

One major challenge to conventional antineoplastic drug therapy is multidrug resistance (MDR), which greatly decreases the efficacy of cancer chemotherapy. Mi et al^[30]^ demonstrated that apatinib increases the effect of paclitaxel against ABCB1 resistant cancer cell xenografts in nude mice. This team also tested whether apatinib would enhance the anticancer effect of doxorubicin on side population phenotype cells and ABCB1-overexpressing MDR leukemia cells by directly suppressing the drug transport function of ABCB1 and increasing the intracellular concentrations of substrate chemotherapeutic drugs.^[31]^ In addition, another study showed that apatinib may restore the sensitivity of lung cancer cells to cisplatin by downregulating MDR1 and inhibiting the activities of proteins in the ERK signaling pathway.^[32]^ In preclinical trials, the synergistic antitumor effect of apatinib was further assessed in combination with docetaxel or doxorubicin and oxaliplatin or 5-FU.^[29]^ However, the ability of apatinib to reverse resistance to cytotoxic chemotherapy agents has not been fully studied in clinical trials.

## 5 Conclusions

In summary, this case report suggests apatinib might be effective for the treatment of primary pleural synovial sarcomas. However, the observed beneficial effect should be verified in further clinical trials. The efficacy of apatinib against other sarcoma subtypes should also be tested.

## Author contributions

**Conceptualization:** Shenglin Ma, Qinghua Deng.

**Project administration:** Sumei Chen.

**Supervision:** Shenglin Ma, Qinghua Deng.

**Writing – original draft:** Sumei Chen.

**Writing – review & editing:** Ke Zhang, Xianqin Wan, Yuanyuan Chen, Qinghua Deng.
